# Prisoners with Attention Deficit Hyperactivity Disorder: Co-morbidities & Service Pathways

**DOI:** 10.1192/j.eurpsy.2022.884

**Published:** 2022-09-01

**Authors:** A. Rawat, E. Chaplin, B. Perera, J. Mccarthy, K. Courtenay

**Affiliations:** 1 WHLDP, CNWL NHS Foundation Trust, Learning Disability, London, United Kingdom; 2 London South Bank University, Medical Sciences, London, United Kingdom; 3 Barnet Enfield and Haringey Mental Health NHS Trust, London, UK, Haringey Ldp, London, United Kingdom; 4 King’s College London, Medical And Health Sciences, London, United Kingdom; 5 Barnet Enfield and Haringey Mental Health NHS Trust, London, UK, Psychiatry - Nlfs, London, United Kingdom

**Keywords:** adhd, Prisoners, Quality Improvement, Service Pathways

## Abstract

**Introduction:**

Effective diagnostic and treatment pathways for ADHD are needed in prison settings due to the high prevalence of ADHD and comorbidities in the prison population.

**Objectives:**

In this presentation, we will describe two studies conducted in seperate London prisons in England. In the first study, the aim was to identify prisoners with ADHD with a focus on describing comorbidity. In the second study, using QI (quality improvement) methodology, the aim was to measure the practicability and effectiveness of a specialist ADHD diagnostic and treatment pathway for prisoners.

**Methods:**

Two studies were carried out in two separate prisons in London. Firstly, data were collected to understand the prevalence of ADHD and the comorbidities. The second study used quality improvement (QI) methodology to assess the impact of a diagnostic and treatment pathway for prisoners with ADHD.

**Results:**

Of the prisoners, 22.5% met the diagnostic criteria for ADHD. Nearly half of them were screened positive for autistic traits, with a higher prevalence of mental disorders among prisoners with ADHD compared to those without. The QI project led to a significant increase in the number of prisoners identified as requiring ADHD assessment but a modest increase in the number of prisoners diagnosed or treated for ADHD.

**Conclusions:**

Despite various challenges, an ADHD diagnostic and treatment pathway was set up in a prison using adapted QI methodology. Further research is needed to explore the feasibility of routine screening for ADHD in prison and examine at a national level the effectiveness of current ADHD prison pathways.

**Disclosure:**

No significant relationships.

